# RNA Polymerase I Is Uniquely Vulnerable to the Small-Molecule Inhibitor BMH-21

**DOI:** 10.3390/cancers14225544

**Published:** 2022-11-11

**Authors:** Ruth Q. Jacobs, Kaila B. Fuller, Stephanie L. Cooper, Zachariah I. Carter, Marikki Laiho, Aaron L. Lucius, David A. Schneider

**Affiliations:** 1 Department of Biochemistry and Molecular Genetics, University of Alabama at Birmingham, Birmingham, AL 35294, USA; 2 Department of Chemistry, University of Alabama at Birmingham, Birmingham, AL 35294, USA; 3 New England Biolabs, Ipswich, MD 01938, USA; 4 Department of Radiation Oncology and Molecular Radiation Sciences and Sidney Kimmel Comprehensive Cancer Center, Johns Hopkins University School of Medicine, Baltimore, MD 21231, USA

**Keywords:** RNA polymerase I, RNA polymerase II, RNA polymerase III, BMH-21, cancer therapeutics, transcription elongation

## Abstract

**Simple Summary:**

Cancer cells upregulate RNA polymerase I (Pol I) activity to increase ribosome abundance in support of rapid cell growth and proliferation. During the last decade, Pol I has emerged as a promising anti-cancer target. Eukaryotes express three closely related RNA polymerases (Pols I, II, and III), responsible for synthesis of all the genome-encoded RNA required by the cell. Effective therapeutic development requires that the treatment be selective for Pol I, without inhibition of Pols II or III. This study evaluates the specificity of the compound BMH-21 on transcription by Pols I, II, and III using purified in vitro transcription assays. These results reveal that Pol I is uniquely sensitive to inhibition by BMH-21, compared to Pols II and III. These findings support ongoing preclinical development of BMH-21 and its derivatives for potential therapeutic applications.

**Abstract:**

Cancer cells require robust ribosome biogenesis to maintain rapid cell growth during tumorigenesis. Because RNA polymerase I (Pol I) transcription of the ribosomal DNA (rDNA) is the first and rate-limiting step of ribosome biogenesis, it has emerged as a promising anti-cancer target. Over the last decade, novel cancer therapeutics targeting Pol I have progressed to clinical trials. BMH-21 is a first-in-class small molecule that inhibits Pol I transcription and represses cancer cell growth. Several recent studies have uncovered key mechanisms by which BMH-21 inhibits ribosome biosynthesis but the selectivity of BMH-21 for Pol I has not been directly measured. Here, we quantify the effects of BMH-21 on Pol I, RNA polymerase II (Pol II), and RNA polymerase III (Pol III) in vitro using purified components. We found that BMH-21 directly impairs nucleotide addition by Pol I, with no or modest effect on Pols II and III, respectively. Additionally, we found that BMH-21 does not affect the stability of any of the Pols’ elongation complexes. These data demonstrate that BMH-21 directly exploits unique vulnerabilities of Pol I.

## 1. Introduction

Eukaryotic RNA polymerases (Pols I, II, and III) [1] are structurally homologous [2,3,4,5,6,7,8,9] but each Pol has unique genetic targets and they each play a distinct role in ribosome biogenesis [10,11]. Pol I transcription of the ribosomal DNA (rDNA) is tightly regulated and proportional to cellular growth rate [12,13]. In human cells, Pol I synthesizes the 47 S pre-ribosomal RNA (rRNA) from clusters of ~400 tandem repeats found on chromosomes 13, 14, 15, 21, and 22 [14,15]. The 47 S pre-rRNA is co- and post-transcriptionally processed to form the mature 18 S, 5.8 S, and 28 S rRNAs [16]. Pol III transcribes the 5 S rDNA genes that are similarly arranged in clusters of tandem repeats on chromosome 1 [17,18]. Finally, Pol II synthesizes the messenger RNA (mRNA) that encodes the 80 ribosomal proteins. All of these components assemble together to form the human ribosome [19].

Most cancer cells require robust ribosome synthesis to support rapid growth and proliferation rates [20]. This “addiction” to ribosome biogenesis represents a unique “Achilles’ heel” in cancer cells [21,22,23]. As a result, several unique ribosome biogenesis inhibitors have been developed to target cancer cells with varying degrees of success [24,25,26,27,28,29,30]. These ribosome biogenesis inhibitors often have off-target effects; they can interfere with mRNA synthesis or translation [31] and induce DNA damage [32,33]. A promising strategy to circumvent these issues is to target Pol I transcription specifically [22,23,34,35]. Because Pol I transcription of the rDNA is the first and rate-limiting step in ribosome biogenesis [36,37], inhibiting Pol I activity will reduce the amount of rRNA available in a cell to form new ribosomes, thereby interfering with cell proliferation. Selective, direct inhibition of Pol I may be challenging due to the structural and functional similarities presented by the three nuclear Pols.

CX-5461 is the first and only Pol I inhibitor to complete Phase I clinical trials [38,39]. Initially, Drygin et al. [39] found that CX-5461 competes with the transcription initiation factor SL1 [40,41] for binding to the rDNA promoter. Its association with the rDNA reduced the binding of SL1 by ~50% and caused a decrease in rRNA synthesis [39]. CX-5461 had an anti-proliferative effect in cancer cell lines, solid tumor cancer cells, and an anti-tumor effect in murine xenograft models [39]. Despite the early promise of CX-5461, in recent years, multiple research groups have shown that CX-5461 does not achieve its chemotherapeutic effects exclusively through Pol I-specific inhibition [33,42]. Xu et al. found that CX-5461 impairs cell growth in malignant cells by stabilizing G-quadruplex DNAs, causing DNA damage [33]. Similarly, Bruno et al. characterized CX-5461 as a topoisomerase II poison, which also results in DNA damage [42]. This was independently confirmed by Pan et al. [43] and is thoroughly reviewed by Xu and Hurley [44]. Considering the recent evidence on CX-5461’s mechanism of action, there remains a need to discover anti-cancer compounds that specifically target Pol I and act independently of DNA damage.

A DNA intercalator, BMH-21, was discovered in a high-throughput cell-based screen for novel p53 activating compounds [45]. It was determined that BMH-21 is a Pol I-inhibitor that does not induce DNA damage, distinguishing it from CX-5461 [29,45,46]. Peltonen et al. found that BMH-21 treatment resulted in potent anti-tumorigenic effects in mammalian cell lines, ex vivo tissues, and mouse models [29,45]. Further mechanistic studies revealed that BMH-21 severely impaired the speed of Pol I transcription elongation in vivo and in vitro, reduced Pol I occupancy of the rDNA, and caused the persistence of long-lived paused Pol I molecules [29,47,48]. Wei et al. found that BMH-21 activates a Pol I regulatory checkpoint that monitors for stalled Pol I molecules and subsequently, triggers the proteasome-mediated degradation of the second largest subunit of Pol I [47]. This regulatory pathway is conserved in both mammalian and *Saccharomyces cerevisiae* (yeast) cells [47]. As a result, yeast is an excellent eukaryotic model for further investigation of the mechanism of BMH-21.

Until this study, the selectivity of BMH-21 inhibition has not been directly tested. It is critical to evaluate this property of BMH-21 as its derivatives progress toward clinical trials. To fill this knowledge gap, we used transient-state kinetics to examine Pol I, II, and III catalyzed transcription elongation in the presence and absence of BMH-21. Even though we know that BMH-21 targets the transcription elongation phase of Pol I transcription [47,48], prior to this study we did not know how BMH-21 influenced the kinetic mechanism of Pol I nucleotide addition. We found that BMH-21 slows Pol I nucleotide addition and induces pausing, ultimately altering the kinetic mechanism of Pol I catalyzed transcription to include pausing pathways. Interestingly, BMH-21 did not affect Pol II elongation under identical experimental conditions. Finally, we observed slight inhibition of Pol III nucleotide addition in the presence of BMH-21 (without induction of a divergent reaction mechanism). Additionally, we found that BMH-21 had no effect on the stability of Pol I, II, and III elongation complexes (ECs). This study provides the first direct evidence that Pol I transcription elongation is uniquely vulnerable to BMH-21 inhibition compared to Pols II and III.

## 2. Materials and Methods

### 2.1. Purification of RNA Polymerases (Pols) I, II, and III

Three *Saccharomyces cerevisiae* (yeast) strains were generated with a C-terminal tag (TEV cleavage site, three HA repeats, and 10 histidine residues) on the second largest subunit of Pol I, II, and III. Yeast were grown at the Bioexpression and Fermentation Facility, Department of Biochemistry and Molecular Biology at the University of Georgia and purified as detailed previously [49,50]. The purified Pol samples were analyzed with SDS PAGE and mass spectrometry [50]. All subunits of Pol I (14-subunits), Pol II (12-subunits), and Pol III (17-subunits) were present with no cross-contamination of subunits across Pol I, II, and III preparations.

### 2.2. Multi-Nucleotide Addition Assay

Multi-nucleotide addition reactions were executed as previously published [49,50,51] and detailed in the Supplemental Materials. Briefly, elongation complexes (ECs) were assembled with purified Pol I, II, or III, pre-annealed RNA:DNA_t_ hybrid, 9-mer RNA annealed to 64-nt DNA template strand, and DNA_nt_ (DNA non-template strand) [52]. EC_9-mer_ were radiolabeled with α-^32^P-CTP in the presence of Mg^2+^ to yield EC_10-mer_. EDTA was added to stop the labeling reaction. Labeled ECs were incubated with BMH-21 (1 μM) or vehicle (BMH-21 storage buffer, 0.1 M NaH_2_PO_4_ pH 6) for 5 min before loading into the rapid mixing instrument, the chemical quenched-flow. A substrate mix including ATP (1 mM), GTP (1 mM), and Mg^2+^ (9 mM) was loaded in the opposite syringe. Radiolabeled ECs and substrates were rapidly mixed together and allowed to incubate for (0.005–10 s). Aliquots of each reaction were run on polyacrylamide sequencing gels to separate starting RNA, 10-mer, from extension products, 11-mer–19-mer.

### 2.3. Analysis of Multi-Nucleotide Addition Data

Multi-nucleotide addition time courses, composed of nine data sets (11-mer–19-mer), were fit globally using a previously published method [49,50,51], Multi-start Evolutionary Nonlinear OpTimizeR (MENOTR) [53]. MENOTR is available on GitHub: https://github.com/ZachIngram/2021-MENOTR, accessed on 22 September 2021. Briefly, MENOTR [53] uses MATLAB (MathWorks, Natick, MA, USA) to combine the genetic algorithm and nonlinear least squares methods to escape local minima and obtain the best parameter estimates for each data set. Replicates were fit individually, and the kinetic parameters of each resulting fit were averaged, and the standard deviation was calculated.

### 2.4. Elongation Complex (EC) Stability Assay

EC stability experiments were executed as previously published [49,50,54,55]. Radiolabeled EC_10-mer_ containing either Pol I, II, and III were mixed with 10 μM RNase A (#LS002132; Worthington Biochemical, Lakewood, NJ, USA), 1 M KCl, and BMH-21 (1 μM) or vehicle at t = 0. EC collapse was determined by calculating the fraction of 7-mer RNA over total RNA.

## 3. Results

### 3.1. BMH-21 Alters the Nucleotide Addition Mechanism of Pol I

**Figure 1:**
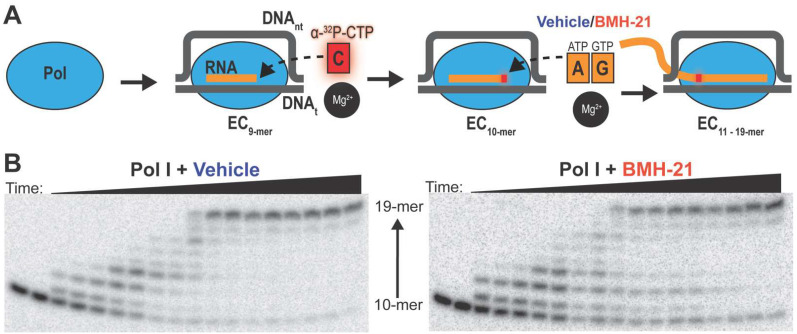
Schematic of multi-nucleotide addition experiment in the presence of vehicle or BMH-21. (**A**) Schematic for multi-nucleotide addition assay. Elongation complexes (ECs) are formed with Pol I, II, or III, RNA:DNA_t_ hybrid, and DNA_nt_. ECs are radiolabeled with α-^32^P-CTP in the presence of Mg^2+^, allowing the formation of a EC_10-mer_. Radiolabeled ECs are incubated with vehicle or BMH-21 (1 µM) for 5 min. ECs are loaded into a chemical quenched-flow instrument and rapidly mixed with ATP (1 mM), GTP (1 mM), and Mg^2+^ (9 mM). Time points are collected over a time course, (0.005–10 s), and run on polyacrylamide sequencing gels to resolve the RNA intermediates, 10-mer–19-mer. (**B**) Representative gels of Pol I multi-nucleotide addition time courses collected in the presence of vehicle, left, or BMH-21 (1 µM), right.

To evaluate the effect of BMH-21 on Pol I transcription elongation, we employed a multi-nucleotide addition assay using oligonucleotide scaffold DNA templates [49,50,51]. Elongation complexes (ECs) were assembled with purified Pol I, a 9-mer RNA pre-annealed to the DNA_t_, and complementary DNA_nt_ strand (Figure 1A). The EC_9-mer_ was radiolabeled with α-^32^P-CTP in the presence of Mg^2+^, yielding EC_10-mer_. The EC_10-mer_ was incubated with BMH-21 (1 µM) or vehicle for 5 min and then loaded into one syringe of a chemical quenched-flow instrument. ATP, GTP, and Mg^2+^ were loaded into the opposite syringe. Based on the template DNA sequence, supplying ATP and GTP allows for nine successive nucleotide incorporation events; 10-mers are extended to full-length 19-mers. ECs and NTPs were rapidly mixed and allowed to incubate for a varying amount of time, (0.005–10 s), before being quenched by 1 M HCl. Each time point was run on a polyacrylamide sequencing gel and exposed to a phosphorimager screen to visualize synthesized RNAs (Figure 1B).

**Scheme 1:**
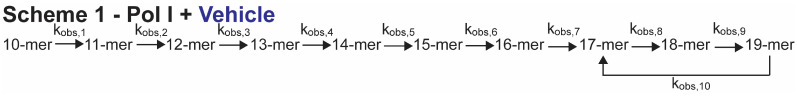
Vehicle-treated Pol I time courses were collected in triplicate and fit to Scheme 1.

**Scheme 2:**
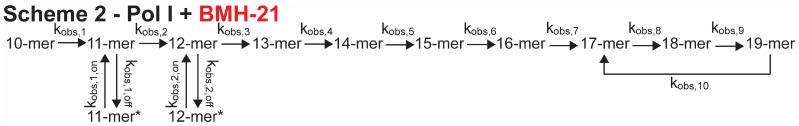
BMH-21-treated Pol I time courses were collected in triplicate and fit to Scheme 2.

**Figure 2:**
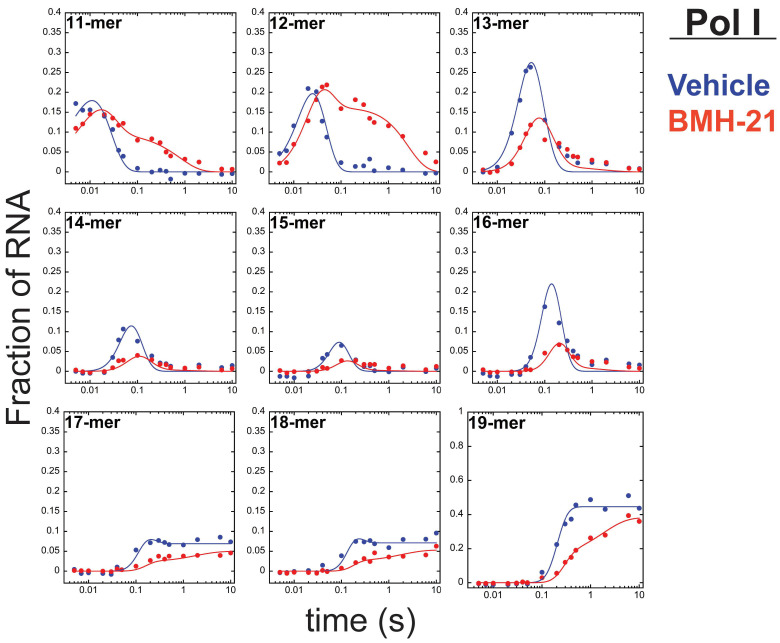
Pol I multi-nucleotide addition in the presence of vehicle or BMH-21. Representative data sets of the fraction of each RNA species over time fit to their respective schemes, Scheme 1 for vehicle-treated and Scheme 2 for BMH-21-treated.

Minimal kinetic models were developed to describe the vehicle- and BMH-21-treated reactions. Experimental data sets consisting of nine time courses, describing the abundance of each RNA intermediate, were fit simultaneously using MENOTR [53]. Vehicle-treated Pol I time courses were fit to Scheme 1, used previously to describe Pol I multi-nucleotide addition [49,50]. Scheme 1 consists of nine individual observed rate constants, k_obs,1_-k_obs,9_, that describe the appearance of each RNA, 11-mer–19-mer. An additional kinetic parameter, k_obs,10_, is required to describe Pol I’s intrinsic nuclease activity. Scheme 1 failed to describe the BMH-21-treated Pol I time courses (Supplemental Figure S1). For the 11-mer and 12-mer, Scheme 1 adequately describes the earlier portion of the intermediate, but unlike the experimental data points, the best fit line sharply falls back to a baseline of 0. For the intermediates 13-mer–16-mer, the best fit line generated by Scheme 1 fails to rise as fast as the experimental data points do. As a result, we determined that BMH-21-treated time courses required a modified scheme, Scheme 2, which includes two pathways leading to and from two paused populations: EC_11-mer*_ and EC_12-mer*_. For each treatment condition, three independent reactions were collected, and each replicate was fit individually. Representative time courses were compared (Figure 2). The mean and standard deviation of each rate constant is reported (Table 1).

The requirement of a modified scheme to describe Pol I multi-nucleotide addition in the presence of BMH-21 is especially apparent at the 11-mer and 12-mer intermediates. In the gels (Figure 1B) and plots (Figure 2), we observed that 11-mers and 12-mers persisted longer in the time course compared to the vehicle treatment. Unlike the single peak observed in the fraction of 11-mer and 12-mer for the vehicle condition, we observed the peak of two distinct populations of 11-mers and 12-mers (Figure 2). This can be explained by Pol I EC_11-mer/12-mer_ existing in two populations: one that is immediately able to undergo nucleotide addition (EC_11-mer/12-mer_), and one that must cycle through an off-pathway step, described by k_obs,1,on_/k_obs,1,off_ and k_obs,2,on_/k_obs,2,off_, before the next nucleotide is added. We describe the intermediate of the off-pathway step as EC_11-mer*_ and EC_12-mer*_ in Scheme 2. From our analysis, we are unable to discern the identity of EC_11-mer*_ and EC_12-mer*_; rather we hypothesize these ECs undergo an extended pause state, and/or a conformational change(s). Additionally, the peak for each intermediate of the 13-mer–19-mer is right-shifted in the presence of BMH-21 compared to the vehicle treatment (Figure 2). This observation indicates slower addition of nucleotides when treated with BMH-21, and indeed, we observed a > 2-fold reduction in the rate constants describing the appearance of the 13-mer and 14-mer (Table 1). Thus, BMH-21 changes the mechanism of Pol I nucleotide addition by inducing pausing and inhibiting nucleotide addition.

### 3.2. Pol II Is Unaffected by BMH-21

**Scheme 3:**
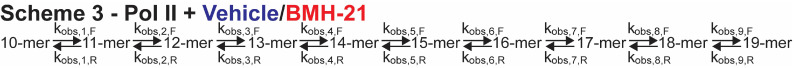
Vehicle- and BMH-21-treated Pol II time courses were collected in triplicate and fit to Scheme 3.

**Figure 3:**
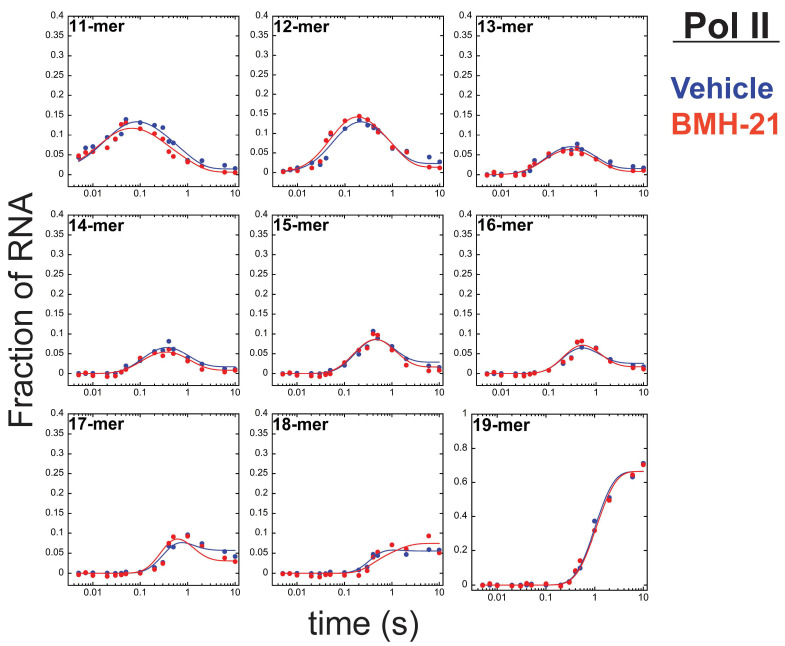
Pol II multi-nucleotide addition in the presence of vehicle or BMH-21. Representative data sets of the fraction of each RNA species over time fit to Scheme 3.

The effect of BMH-21 on Pol II was very different than on Pol I. Vehicle- and BMH-21-treated Pol II time courses were collected and fit to Scheme 3. Pol II multi-nucleotide addition in the presence of vehicle and BMH-21 required forward (k_obs,F_) and reverse (k_obs,R_) rate constants between each RNA intermediate. Our data suggests that pyrophosphate is slow to release from the active center, which allows for the reverse reaction of nucleotide addition, pyrophosphorolysis, to occur (unpublished). Vehicle and BMH-21 representative data were compared (Figure 3). For each treatment condition, three independent reactions were collected, and rate constants were calculated and reported (Supplementary Table S1). To facilitate straightforward comparisons of the vehicle- and BMH-21-treated reactions, we calculated and reported the ratio of the forward and reverse rate constants (Table 2). Resultant ratios were within error between the vehicle- and BMH-21-treated data sets. It is evident from both the raw data (Figure 3) and the fit parameters (Table 2) that BMH-21 has no effect on Pol II transcription elongation.

### 3.3. BMH-21 Has a Modest Inhibitory Effect on Pol III

**Scheme 4:**
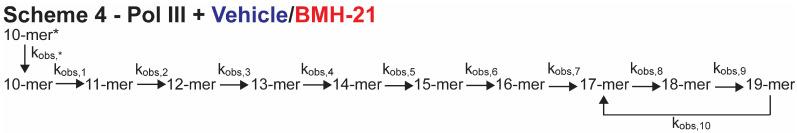
Vehicle- and BMH-21-treated Pol III time courses were collected in triplicate and fit to Scheme 4.

**Figure 4:**
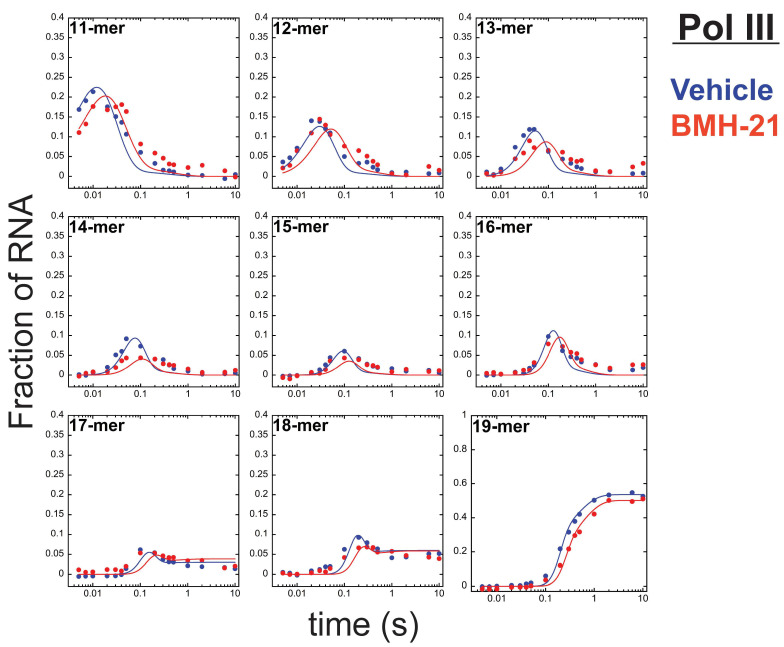
Pol III multi-nucleotide addition in the presence of vehicle or BMH-21. Representative data sets of the fraction of each RNA species over time fit to Scheme 4.

Finally, we investigated the effect of BMH-21 on Pol III transcription elongation. Pol III time courses were fit similarly to Pol I vehicle-treated time courses. The only difference was the requirement for inclusion of an additional kinetic parameter, k_obs,*_, that describes the conversion of an inactive subpopulation of Pol III ECs (EC_10-mer*_) to an elongation competent conformation (EC_10-mer_), shown in Scheme 4. Rate constants k_obs,1_–k_obs,9_ describe the appearance of each RNA intermediate while k_obs,10_ describes Pol III’s intrinsic nuclease activity. Representative vehicle- and BMH-21-treatment data sets were compared (Figure 4). For each treatment condition, three independent reactions were collected, and the average and standard deviation of each rate constant are reported (Table 3).

We observed a small, but clear right-shift in the time courses in the presence of BMH-21 compared to the vehicle (Figure 4). This observation indicates that BMH-21 decreases the rate of nucleotide addition by Pol III at one or more steps in the multi-nucleotide addition reaction which results in a ~20% reduction of the average k_obs_ from ~49 s^−1^ (vehicle) to ~39 s^−1^ (BMH-21) (Table 3). Despite the decrease in some of the observed rate constants, BMH-21- and vehicle-treated data sets did not require an alternative reaction scheme, as was observed for Pol I. Reduced rate constants for individual steps in nucleotide addition demonstrate that BMH-21 has a slight, but observable inhibitory effect on Pol III transcription.

### 3.4. BMH-21 Does Not Destabilize Pol I, II, or III Elongation Complexes

**Figure 5:**
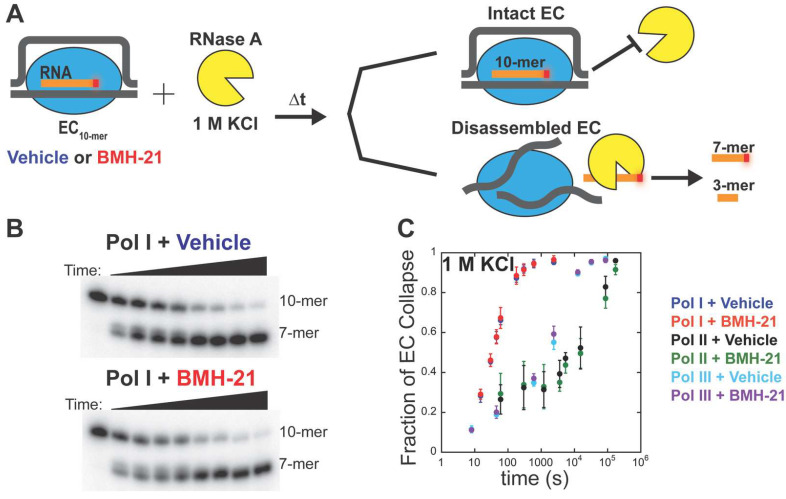
Pol I, II, and III EC stability in the presence of vehicle or BMH-21. (**A**) Schematic for EC stability assay. Pol I, II, or III radiolabeled ECs are mixed with vehicle or BMH-21 (1 µM), RNase A (10 µM), and KCl (1 M) at t = 0. Reactions were collected continuously and quenched in loading dye. Reactions were run on polyacrylamide sequencing gels to resolve the 10-mers and 7-mers that evidence intact or collapsed ECs, respectively. (**B**) Representative gels of Pol I EC stability time courses collected in the presence of vehicle, top, or BMH-21, bottom. (**C**) Plot of the fraction of EC collapse over time for each Pol in presence of vehicle and BMH-21. Time courses were collected in triplicate and the mean value is plotted with error bars corresponding to the standard deviation about the mean.

Previous studies show that BMH-21 causes Pol I pausing/arrest [47,48] and a decrease in occupancy of the rDNA [48]. Consistently, we observed a persistence of the 11-mer and 12-mer in the presence of BMH-21, which could also be due to pausing/arrest. It is possible that this is achieved by destabilizing the ECs, which results in the eviction of some Pol I molecules from the DNA. To test this hypothesis, we employed an EC stability assay [49,50,54] (Figure 5A). Pol I, II, or III radiolabeled ECs were incubated in high salt conditions (1 M KCl), RNase A (10 µM), and BMH-21 (1 µM) or vehicle at t = 0. Time points were collected to monitor EC collapse over time. If an EC is intact at a given time point, the EC protects the 10-mer from RNase A cleavage, and 10-mer RNA is detected on the gel (Figure 5B). If the EC has disassembled, RNase A will cleave the exposed 10-mer and we will observe a 7-mer (Figure 5B). The fraction of 7-mer over total RNA was calculated at each time point for Pols I, II, and III over three independent reactions. We plotted EC disassembly over time and found that the stability of Pol I, II, and III ECs were unaffected by BMH-21 treatment (Figure 5C). This result affirms that BMH-21 does not have off-target effects on Pol II and III stabilities, and that BMH-21 does not reduce rRNA synthesis through eviction of Pol I molecules from the template DNA.

## 4. Discussion

### 4.1. BMH-21 Significantly Impairs Pol I Transcription Elongation

In this study, we have shown that BMH-21 perturbs the reaction scheme for Pol I transcription elongation, while BMH-21 modestly inhibited Pol III and had no effect on Pol II. These in vitro results do not exclude the possibility that BMH-21 may influence Pol II transcription at some genetic loci in vivo, especially at G-rich loci; however, this work demonstrates that Pol II is inherently less sensitive to the effects of BMH-21. Our EC stability results indicate that BMH-21 does not destabilize Pol I, II, or III ECs. Our results are compatible with previous in vivo observations [47,48]. Together, these in vitro findings explain how BMH-21 activates a Pol I regulatory checkpoint: BMH-21 induces pause pathways that increase the amount of stalled Pol I ECs [29,47,48] which results in their ubiquitination (Pitts, et. al, accepted) and subsequent degradation in vivo [47] in a Pol I-specific manner.

### 4.2. BMH-21’s Modest Inhibitory Effect on Pol III May Be Therapeutically Advantageous

Here, we revealed that both transcription elongation by Pol I and Pol III are inhibited by BMH-21. While Pol I is most severely inhibited, the modest effect on Pol III is an important discovery. The rationale for targeting Pol I for therapeutic benefit is similar to the rationale for targeting Pol III [56]. Like Pol I, Pol III transcription is tightly linked to translation levels [57]. Pol III synthesizes 5 S rRNA, essential for the formation of the large ribosomal subunit, and transfer RNA (tRNA), RNA molecules that serve as adapters during translation of mRNA by the ribosome. Similarly to Pol I, Pol III transcription is dysregulated in cancer cells [58,59] which results in increased levels of 5 S rRNAs and tRNAs [60,61]. Ultimately, we know that the dysregulation of Pol III transcription is a requirement for malignant cells to promote tumorigenesis [62]. Therefore, it is possible that while BMH-21 is preferentially selective for Pol I, partial inhibition of Pol III transcription could enhance its anti-cancer effects.

### 4.3. Intrinsic Biochemical Properties of the Pols Render Them More or Less Sensitive to BMH-21

While Pols I, II, and III are structurally homologous enzymes, we have shown that Pol I is the most sensitive to BMH-21 treatment in vitro. How can this be explained? Our recent comparisons of the Pols suggests that over the course of evolution, selective pressures unique to each Pol have led to three biophysically distinct molecular machines [49,50]. We hypothesize that their unique enzymatic properties render them more or less vulnerable to BMH-21. Previously, we revealed that nucleotide addition rate constants describing Pol I, II, and III transcription elongation varied substantially [49,50]. Pol I is the fastest, followed by Pol III, and Pol II is the slowest. Interestingly, we found that while Pol I is capable of the fastest elongation kinetics of the Pols, it is also the most sensitive Pol to EC collapse, identity of encoded nucleotides, and alterations in reaction conditions [50]. Taken together, we theorize that BMH-21 intercalation inhibits Pol I most severely by exploiting Pol I’s high sensitivity to perturbations of the DNA environment, relative to the other Pols.

## 5. Conclusions

In conclusion, there is a need to define novel cancer therapeutics that are selective for Pol I transcription. Preclinical studies show that BMH-21 is a promising anti-cancer compound and its derivatives are progressing toward clinical trials. Here, we showed that Pol I is the most vulnerable Pol to BMH-21, exemplified by a reduction in elongation rate and induction of pausing pathways. While BMH-21 slowed Pol III elongation, its effect did not fundamentally alter the reaction scheme. Finally, Pol II was unaffected by BMH-21. This work supports BMH-21’s continued preclinical development, ultimately empowering selective inhibition of ribosome biosynthesis in a variety of cancer types.

## Figures and Tables

**Table 1:** Resultant parameter values from Pol I vehicle- and BMH-21-treated multi-nucleotide time courses.

Kinetic Parameter (s^−1^)	Vehicle ^a^	BMH-21 (1 µM) ^b^
k_obs,1_	80 ± 4	60 ± 20
k_obs,2_	120 ± 30	90 ± 20
k_obs,3_	66 ± 6	30 ± 7
k_obs,4_	26 ± 3	13 ± 3
k_obs,5_	53 ± 9	40 ± 20
k_obs,6_	63 ± 5	40 ± 20
k_obs,7_	17 ± 4	13 ± 3
k_obs,8_	48 ± 5	70 ± 50
k_obs,9_	52 ± 3	60 ± 40
k_obs,10_	8 ± 2	8 ± 7
k_obs,1,on_	-	2.5 ± 0.8
k_obs,1,off_	-	25 ± 5
k_obs,2,on_	-	0.9 ± 0.4
k_obs,2,off_	-	17 ± 5

^a^ Vehicle-treated time courses were fit to Scheme 1. Pol I multi-nucleotide addition time courses were collected in triplicate and globally fit. The resultant mean and standard deviation of the optimized parameters are reported. ^b^ BMH-21-treated time courses were fit to Scheme 2. Pol I multi-nucleotide addition time courses were collected in triplicate and globally fit. The resultant mean and standard deviation of the optimized parameters are reported.

**Table 2:** Ratios of forward and reverse parameter values from Pol II vehicle- and BMH-21-treated multi-nucleotide time courses.

Kinetic Parameter	Vehicle ^a^	BMH-21 (1 µM) ^b^
k_obs,1,F_/k_obs,1,R_	0.43 ± 0.06	0.5 ± 0.1
k_obs,2,F_/k_obs,2,R_	1.9 ± 0.6	5 ± 3
k_obs,3,F_/k_obs,3,R_	0.8 ± 0.2	1.0 ± 0.5
k_obs,4,F_/k_obs,4,R_	1.4 ± 0.5	1.6 ± 0.5
k_obs,5,F_/k_obs,5,R_	1.8 ± 0.3	2.6 ± 0.7
k_obs,6,F_/k_obs,6,R_	0.92 ± 0.04	1.5 ± 0.6
k_obs,7,F_/k_obs,7,R_	2.0 ± 0.2	2.6 ± 0.7
k_obs,8,F_/k_obs,8,R_	1.0 ± 0.3	1.7 ± 0.7
k_obs,9,F_/k_obs,9,R_	20 ± 20	20 ± 10

^a^ Vehicle-treated time courses were fit to Scheme 3. Pol II multi-nucleotide addition time courses were collected in triplicate and globally fit. The resultant mean and standard deviation of the optimized parameters are reported. ^b^ BMH-21-treated time courses were fit to Scheme 3. Pol II multi-nucleotide addition time courses were collected in triplicate and globally fit. The resultant mean and standard deviation of the optimized parameters are reported.

**Table 3:** Resultant parameter values from Pol III vehicle- and BMH-21-treated multi-nucleotide time courses.

Kinetic Parameter (s^−1^)	Vehicle ^a^	BMH-21 (1 µM) ^b^
k_obs_,_*_	1.8 ± 0.6	2.1 ± 0.5
k_obs,1_	138 ± 7	104 ± 3
k_obs,2_	46.3 ± 0.5	30 ± 4
k_obs,3_	50 ± 1	25 ± 3
k_obs,4_	40 ± 2	31 ± 5
k_obs,5_	44 ± 2	54 ± 5
k_obs,6_	60 ± 1	54 ± 9
k_obs,7_	26 ± 1	20 ± 2
k_obs,8_	54 ± 3	46 ± 4
k_obs,9_	31 ± 3	30 ± 3
k_obs,10_	3.5 ± 0.5	3.3 ± 0.4

^a^ Vehicle-treated time courses were fit to Scheme 4. Pol III multi-nucleotide addition time courses were collected in triplicate and globally fit. The resultant mean and standard deviation of the optimized parameters are reported. ^b^ BMH-21-treated time courses were fit to Scheme 4. Pol III multi-nucleotide addition time courses were collected in triplicate and globally fit. The resultant mean and standard deviation of the optimized parameters are reported.

## Data Availability

Further information and requests for data should be directed to and will be fulfilled by David Schneider (dschneid@uab.edu).

## Supplementary Materials

The following supporting information can be downloaded at: https://www.mdpi.com/article/10.3390/cancers14225544/s1, Figure S1: Pol I multi-nucleotide addition in the presence of BMH-21. Representative data set of each RNA species over time fit to Scheme 1; Table S1: Resultant parameter values from Pol II vehicle- and BMH-21-treated multi-nucleotide time courses fit to Scheme 3; Detailed Materials.

## Abbreviations

Pol I	RNA polymerase I
Pol II	RNA polymerase II
Pol III	RNA polymerase III
rDNA	ribosomal DNA
rRNA	ribosomal RNA
mRNA	messenger RNA
EC	elongation complex
DNA_t_	DNA template
DNA_nt_	DNA non-template
MENOTR	Multi-start Evolutionary Nonlinear OpTimizeR
tRNA	transfer RNA
