# Supplementary material for: RNA Polymerase I Is Uniquely Vulnerable to the Small-Molecule Inhibitor BMH-21
Source: Cancers (Basel). 2022 Nov 11;14(22):5544. doi: 10.3390/cancers14225544 (PMC9688676; doi:10.3390/cancers14225544)
Supplement: Supplementary file 1 [file cancers-14-05544-s001.zip › cancers-2019374-supplementary.pdf]

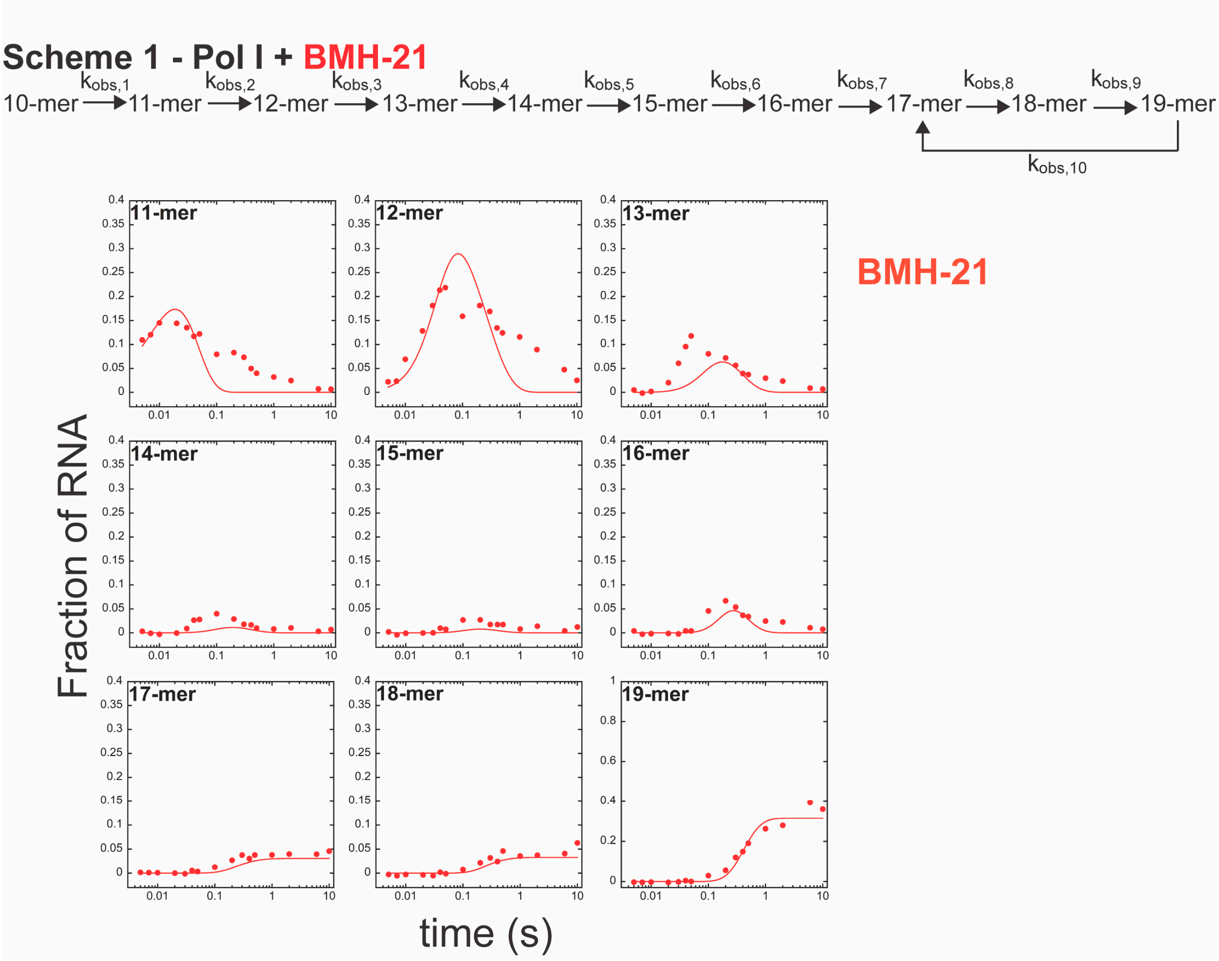

Supplementary Figure S1. Pol I multi-nucleotide addition in the presence of BMH-21. Representative data set of each RNA species over time fit to Scheme 1.

Kinetic Parameter (s^{-1})	Vehicle[a]	BMH-21 (1 μM)[b]
$k_{obs,1,F}$	10 ± 3	12 ± 3
$k_{obs,1,R}$	24 ± 3	27 ± 8
$k_{obs,2,F}$	30 ± 3	30 ± 10
$k_{obs,2,R}$	17 ± 6	9 ± 8
$k_{obs,3,F}$	34 ± 9	19 ± 9
$k_{obs,3,R}$	50 ± 20	20 ± 20
$k_{obs,4,F}$	50 ± 20	50 ± 30
$k_{obs,4,R}$	40 ± 30	40 ± 30
$k_{obs,5,F}$	40 ± 20	30 ± 20
$k_{obs,5,R}$	20 ± 10	10 ± 10
$k_{obs,6,F}$	30 ± 10	20 ± 10
$k_{obs,6,R}$	40 ± 20	10 ± 10
$k_{obs,7,F}$	23 ± 8	15 ± 5
$k_{obs,7,R}$	12 ± 6	7 ± 5
$k_{obs,8,F}$	400000 ± 900000	11 ± 5
$k_{obs,8,R}$	600000 ± 900000	8 ± 8
$k_{obs,9,F}$	10 ± 4	10 ± 9
$k_{obs,9,R}$	0.8 ± 0.5	0.94 ± 0.02

Supplemental Table S1. Resultant parameter values from Pol II vehicle- and BMH-21-treated multi-nucleotide time courses fit to Scheme 3.

[a]Vehicle-treated time courses were fit to Scheme 3. Pol II multi-nucleotide addition time courses were collected in triplicate and globally fit to their respective schemes. The resultant mean and standard deviation of the optimized parameters are reported. [b] BMH-21-treated time courses were fit to Scheme 3. Pol II multi-nucleotide addition time courses were collected in triplicate and globally fit to their respective schemes. The resultant mean and standard deviation of the optimized parameters are reported.

Detailed Materials

Buffers: All buffers were made with Millipore filtered deionized water and filtered using Millipore 0.22 μm filters (MilliporeSigma, Billerica, MD). Transcription reactions proceeded in buffer A [52]: 40 mM KCl, 20 mM Tris-Acetate (OAc) pH 7.9 at 25 °C, 2 mM dithiothreitol, 0.2 mg/ml^{-1} bovine serum albumin.

Proteins: Pols I, II, and III were purified from *Saccharomyces cerevisiae* as detailed previously [49, 50]. The identities of Pol fractions were verified with Coomassie Blue SDS PAGE, western blots, and mass spectrometry [50]. RNase A (catalog # LS002132; Worthington Biochemical, Lakewood, NJ) was dialyzed into buffer A with 20% glycerol. Concentration was determined by spectroscopic assay in denaturing protein measurement buffer as previously described [54].

Nucleic acids: The following nucleic acids were purchased from Integrated DNA Technologies (Cedar Rapids, IA):
DNA non-template strand (DNA_{nt}) 5′
ACCAGCAGGCCGATTGGGATGGGTATTCCCTCCTGCCTCTCGATGGCTGTAAGTATCCTATAGG
RNA 5′-AUCGAGAGG
DNA template strand (DNA_t) 5′
CCTATAGGATACTTACAGCCATCGAGAGGCAGGAGGGAATACCCATCCCAATCGGCCTGCTGGT

NTP substrates: Adenosine triphosphate (ATP) and guanosine triphosphate (GTP) were purchased from Sigma Aldrich as lyophilized sodium salts. ATP and GTP salts were dissolved in buffer A, filtered with Millipore 0.22 μm filters, and dialyzed into buffer A as previously detailed [52].

Chemical quenched-flow time courses: For multi-nucleotide addition experiments, two solutions were rapidly mixed together in the instrument: EC mix and NTP mix. The EC mix contained ~ 16 nM Pol I, 162.75 nM RNA, 54.26 nM DNA_t, 162.75 nM DNA_{nt}, 5 nM α-^{32}P-CTP, 100 μM $Mg(OAc)_2$, and 1.1 mM EDTA. The NTP mix contained 2 mM ATP, 2 mM GTP, 18 mM $Mg(OAc)_2$, and 0.05 mg/mL^{-1} heparin. The EC mix and NTP mix are mixed 1:1, allowed to incubate for a varying amount of time (0.005 – 10 s) before being quenched in 1 M HCl. An aliquot of the quenched reaction sample is mixed with equalizing HCl and neutralization buffer.

Gel electrophoresis: RNAs produced from multi-nucleotide addition and EC stability experiments were separated by denaturing polyacrylamide sequencing gel electrophoresis. Samples were boiled at 95 ºC for 5 min and ran on a 28 % acrylamide (19:1, acrylamide:bis-acrylamide), 7 M urea gel in 1X TBE.
